# The Reporter System for GPCR Assay with the Fission Yeast *Schizosaccharomyces pombe*

**DOI:** 10.6064/2012/674256

**Published:** 2012-12-31

**Authors:** Shintaro Sasuga, Toshiya Osada

**Affiliations:** Department of Life Science, Graduate School of Bioscience and Biotechnology, Tokyo Institute of Technology, B-2 4259 Nagatsuta-cho, Midori-ku, Yokohama 226-8501, Japan

## Abstract

G protein-coupled receptors (GPCRs) are associated with a great variety of biological activities. Yeasts are often utilized as a host for heterologous GPCR assay. We engineered the intense reporter plasmids for fission yeast to produce green fluorescent protein (GFP) through its endogenous GPCR pathway. As a control region of GFP expression on the reporter plasmid, we focused on seven endogenous genes specifically activated through the pathway. When upstream regions of these genes were used as an inducible promoter in combination with LPI terminator, the *mam2* upstream region produced GFP most rapidly and intensely despite the high background. Subsequently, LPI terminator was replaced with the corresponding downstream regions. The SPBC4.01 downstream region enhanced the response with the low background. Furthermore, combining SPBC4.01 downstream region with the sxa2 upstream region, the signal to noise ratio was obviously better than those of other regions. We also evaluated the time- and dose-dependent GFP productions of the strains transformed with the reporter plasmids. Finally, we exhibited a model of simplified GPCR assay with the reporter plasmid by expressing endogenous GPCR under the control of the foreign promoter.

## 1. Introduction

In mammalians, G protein-coupled receptors (GPCRs) constitute the largest and most divergent protein families. GPCRs are activated by hormones, odorants, peptides, neurotransmitters, and so on [1–3]. Not surprisingly, it is often the case that GPCRs are associated with various diseases; GPCRs are one of the most potential drug targets, along with enzymes. However, it is difficult to reproduce appropriate and functional GPCR expression in heterologous cells due to the distinctive conformation and unknown mechanism of its trafficking and folding. Therefore, a large number of GPCRs still do not determine the corresponding ligands. Today, a wide variety of hosts and their transformants have been developed to resolve these problems [4–10].

Fission yeast, *Schizosaccharomyces pombe*, is a unicellular eukaryote and the best model organism especially in cell cycle. Fission yeast shares more similarities to mammals in terms of mRNA splicing, posttranslational modification, and so on than other yeasts including budding yeast, *Saccharomyces cerevisiae* [11]. Indeed, many genes of fission yeast can be complemented by the mammalian homologs, and many mammalian proteins are successfully expressed in fission yeast cells [12–17]. Although fission yeast has so many advantages in expressing heterologous protein, it is preferable not to select fission yeast in the GPCR study.

Fission yeast endogenously follows two alternative GPCR pathways. One is the nutrient (glucose) signaling pathway [18] and the other is the mating pathway. This mating pathway closely resembles the mitogen-activated protein kinase (MAPK) pathway in mammalian cells [19, 20]. A fission yeast cell usually divides by mitosis in rich medium. However, exposed to nutrient starvation (particularly nitrogen starvation), the cell converts mitosis to meiosis by which the cell develops into robust spores with opposite mating type via pheromone communication for resistance to environmental stress [20, 21]. One mating type, plus cell (*h*+ or P cell), secretes diffusible peptide pheromone, which is P-factor encoded by *map2* gene. Another mating type, minus cell (*h*− or M cell), receives P-factor by the endogenous GPCR, which is Mam2 protein encoded by *mam2* gene [22, 23]. The pheromone reception triggers sequential and synergistical activation of mating specific genes, which results in morphological changes for later conjugation to opposite mating type [23, 24]. 

In this study, we proposed the screening system with fission yeast through the endogenous mating pathway to find the ligand of a number of orphan GPCRs. As a reporter gene, the intense genes such as LacZ or luciferase are often utilized in GPCR assay with yeasts [8–10, 25], but we used green fluorescent protein (GFP) with a focus on easy and inexpensive detection in spite of a very weak signal compared to that of intense genes. In order to overcome this disadvantage, we constructed the tractable reporter plasmids.

## 2. Materials and Methods

### 2.1. Strains and Media

The strains used in this study are listed in Table 1. The fission yeast cells were grown in EMM (Edinburgh Minimal Medium, Sunrise Science Products, CA, USA) or EMM-N (EMM minus Nitrogen, Sunrise Science Products). Transformants were plated onto MMA (Minimal Medium Agar, Sunrise Science Products) or MMA supplemented with 1.25% leucine (120 *μ*L/plate).

*Escherichia coli* strain DH5*α* was used for the subcloning of the plasmid preparation.

Peptide and oligonucleotide synthesis were performed by Operon Co. Ltd. (Tokyo, Japan). Each peptide was prepared as a stock solution of 1 mM in Milli-Q water and stored at −80°C.

### 2.2. Plasmid Constructions and Transformations

#### 2.2.1. Gene Disruption

The gene disruption was performed by standard homologous recombination method. The detailed construction of the plasmids for gene disruption was described previously [26]. Briefly, about 1000 bp of 5′ and 3′ flanking sequences of a target gene were used as the chromosomal integration regions. To delete the *ura4* selection marker, the *ura4* gene was sandwiched with about 200 bp of 3′ flanking sequence of the target gene. For negative selection of the *ura4*-cells, the cells were plated onto the YES-FOA plates (0.5% Yeast Extract, 3% glucose and SP Supplements, 2% Bacto agar, 0.1% 5-fluoroorotic acid). The resultant *ura4*-cells were available for the subsequent gene recombination.

#### 2.2.2. Reporter Plasmids Based on pAL7

**Figure 1:**
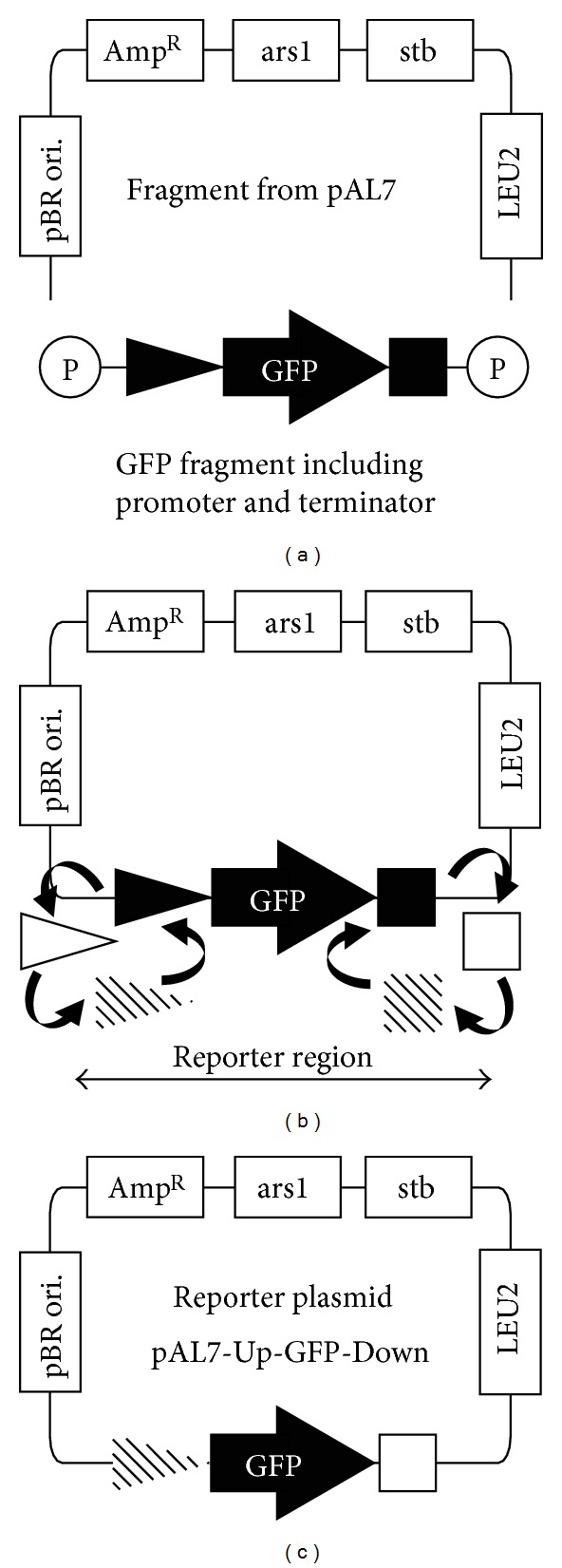
The schematic illustration of reporter plasmid construction. (a) Ligation of the fragment amplified from pAL7 and the phosphated fragment including a series of promoter, GFP (filled arrow) and LPI terminator (filled box). (b) Conversion of the promoter into any upstream regions (shaded and open triangles), and conversion of the LPI terminator into any downstream regions (shaded and open boxes). (c) Completion of converting upstream and downstream region. All reporter plasmids listed in Table 1 were constructed in this way.

The reporter plasmids were engineered based on pAL7 which was a high copy plasmid for fission yeast [27]. The schematic illustration was described in Figure 1. The main fragment including the replication origin and selection marker was amplified from pAL7 using primers pAL7invforward and pAL7invreverse. The fragment was ligated with a series of a certain promoter, GFP, and Lipocortin I (LPI) terminator. To replace the promoter region with each upstream region of the pheromone-dependent gene, inverse PCR was performed from the resultant plasmid using primers pAL7invreverse and GFPORFforward. To replace the LPI terminator, inverse PCR was performed using primers pAL7invforward and GFPORFreverse. Open reading frame (ORF) of GFP used in this study was amplified from a Monster Green Fluorescent Protein phMGFP Vector (Promega Japan, Tokyo, Japan). LPI terminator was amplified from a pSU1Z vector (Asahi Glass Co., Ltd, Tokyo, Japan). Upstream and downstream regions were amplified from a genomic DNA, and the primers were listed in Table 3.

#### 2.2.3. *mam2* Gene Expression Plasmid

pSU1Z vector allows the expression of particular genes under the control of the hCMV promoter at the *ura4* locus on the fission yeast chromosome. To express *mam2* gene under the control of other promoters, we replaced hCMV promoter with *nmt1* or *urg1* promoter. The region of *nmt1* promoter was referred to pREP1 vector and that of *urg1* was referred to the region reported by Watt et al. [28]. To repress the *nmt1* promoter, more than 15 *μ*M of thiamine were added to the medium. To induce the *nmt1* promoter, the cells were incubated in fresh EMM without thiamine for 20 hours. During the pre-incubation, *urg1* promoter was consistently repressed by the absence of uracil. On the contrary, *urg1* promoter was constitutively activated during the assay by the nitrogen starvation.

All PCR products used for the plasmid construction were prepared using the KOD-plus-Neo (Toyobo, Osaka, Japan) in accordance with the supplier's instructions. All fragments without the replication origin of *Escherichia coli* were phosphorylated with T4 Polynucleotide Kinase (Toyobo) and the ligation reactions were performed with a Ligation-Convenience Kit (Nippon gene, Tokyo, Japan). The sequence of each plasmid was verified by the nucleotide sequence analysis.

#### 2.2.4. Transformation

The fission yeast was transformed using a lithium acetate method [27, 29]. Transformed cells were plated onto MMA plates or MMA plates supplemented with leucine. The plates were incubated at 32°C for 2-3 days, and positive colonies were selected. To check for correct integration, PCR was performed on the extracted DNA using SapphireAmp Fast PCR Master Mix (Takara Bio Inc., Otsu, Japan). All the parental strains in this study lacked the function of both *leu1*+ and *ura4*+. Before assay, *leu1-32* was complemented with LEU2 derived from pAL7 (or its derivatives) and *ura4-D18* was complemented with *ura4* gene derived from pSU1Z vector (or its derivatives).

### 2.3. Assay

#### 2.3.1. Time-Dependent Assay

The cells were grown in EMM at 32°C for 24–36 hours and were inoculated into 5 mL of the fresh EMM. Then the cells were grown at 30°C for 20 hours and harvested. After washing twice with sterile water, the cells were transferred to assay medium to give an initial optical density of 1.5 at 600 nm. Immediately, P-factors were added at a final concentration of 1 or 0 *μ*M. The cells in 500 *μ*L were taken every hour (0–6 h), two hours (6–12 h), or twelve hours (12–24 h). Aliquots were resuspended in 500 *μ*L of Milli-Q water after being washed once. Fluorescence intensity of GFP produced in the cells was measured by a Hitachi F-2700 Fluorescence Spectrophotometer (Hitachi, Tokyo Japan). The relative fluorescence intensity was averaged and calculated as the ratio of the sample to the control strain harboring pAL7 instead of the reporter plasmid (OSP210-0 or OSP220-0). The images were taken on a Zeiss Axiovert 200M Inverted Microscope with the AxioCam MRm charge-coupled camera controlled by Axiovision. Assays were performed using independent transformants.

#### 2.3.2. Dose-Dependent Assay

After preincubation, the cells were transferred to EMM-N to give an initial optical density of 1.5 at 600 nm. The cells exposed to each concentration of P-factor were incubated at 30°C for 24 hours, and the fluorescence intensity was measured. Normalized data were fit to the equation: *E* = *E*_max⁡_/(1 + exp⁡⁡[*γ*∗(ln⁡[EC_50_] − ln⁡[*x*])]). *E* represents the current response at each concentration of P-factor, *x*. *E*_max⁡_ is the maximal response. EC_50_ is the concentration of P-factor yielding a half maximal response. *γ* is the Hill coefficient.

## 3. Results

### 3.1. Tractable Reporter Plasmid Developed from pAL7

To prepare the transformants for reporter assay, we constructed the new reporter plasmids with both tractability and sensitivity. The reporter plasmids made those transformants produce green fluorescent protein (GFP) through the Mam2-P-factor signal transduction pathway (mating pathway). To determine the most appropriate pheromone-dependent reporter region on the reporter plasmid, we focused on the 7 genes, which were previously reported to have been sharply activated by the addition of P-factor [24, 30]. The upstream regions of those genes were used as an inducible promoter for GFP. As the downstream region of GFP, we employed LPI terminator, which correctly worked as a gene terminator in the fission yeast cell. Each of the resultant plasmids (listed in Table 2) was transformed into the *sxa2*Δ strain, OSP210 (*h*−, *leu1-32*, *ura4-D18*, *sxa2*Δ). The reason why *sxa2* gene was deleted was because Sxa2 protein was the specific peptidase of P-factor. The response to the 1 or 0 *μ*M of P-factor under nitrogen starvation was shown in Table 2. All upstream regions other than the *dhc1* upstream region expressed GFP. The *mam2* upstream region responded much more intensely than others. Nevertheless, the signal to noise ratio (SNR) of *mam3* and *sxa2* upstream regions was better than that of the *mam2* upstream region. Next, we determined whether some downstream regions were more suitable for 3′ UTR of GFP than the LPI terminator since it was reported that 3′ UTR of mRNA regulated its stability and had an effect on the expression level [31]. LPI terminator in the reporter region of each plasmid was replaced with the downstream region corresponding to its upstream region to reproduce the native genomic context. The combination of *dhc1* regions did not respond, suggesting that the important region for transcription might locate on ORF or the outside of the regions used in this study. The *spk1* downstream region decreased the background under the absence of P-factor. The *mam2* and *mam3* downstream regions obviously decreased the response to P-factor, while that of *sxa2* showed little difference from LPI terminator. The SPBC4.01 and *rgs1* downstream regions raised the response dramatically, but it was clear that the *rgs1* downstream region had raised the expression level without relying on P-factor. In an effort to prepare the appropriate reporter region, the *mam2*, *mam3,* and *sxa2* upstream regionS presumably including the intense pheromone-dependent upstream activation sequence were combined with SPBC4.01 downstream region. As we had expected, the combination of the *sxa2* upstream and SPBC4.01 downstream regions had raised the response with little increase of background. Thereby, the best SNR was obtained with this combination. We circumstantially investigated the two reporter plasmids; one is pAL7-U*mam2*-GFP-LPI including the most intense reporter region and the other is pAL7-U*sxa2*-GFP-DSPBC4.01 whose reporter region exhibited the best SNR.

**Figure 2:**
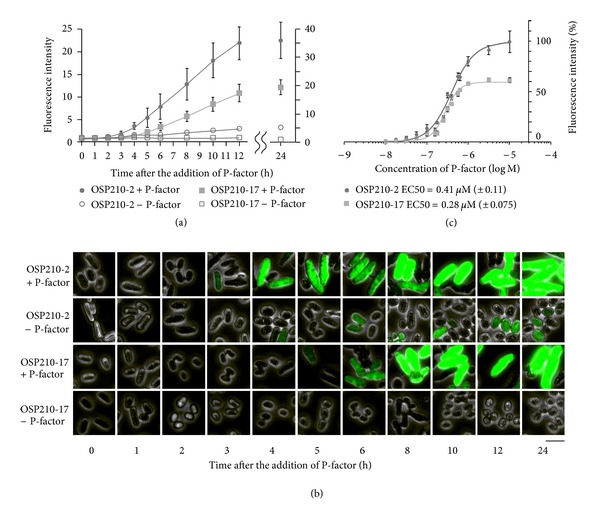
Time- and dose-dependent response to P-factor with the reporter plasmids. (a) Time-dependent response to P-factor with either pAL7-U*mam2*-GFP-LPI or pAL7-U*sxa2*-GFP-DSPBC4.01. The cells were exposed to 1 or 0 *μ*M of P-factor and were taken every hour (0–6 h), two hours (6–12 h) or twelve hours (12–24 h). Filled circle depicts OSP210-2 (*sxa2*Δ, pAL7-U*mam2*-GFP-LPI) +P-factor, open circle depicts OSP210-2 −P-factor, filled square depicts OSP210-17 (*sxa2*Δ, pAL7-U*sxa2*-GFP-DSPBC4.01) +P-factor and open square depicts OSP210-17 −P-factor. The values were the means of triplicate determinations from a typical experiment. The error bars represent ± standard error. (b) Aliquots of culture medium (2 *μ*L) were mounted on slides and visualized by a fluorescence microscope. After the addition of P-factor, the morphology was gradually changed and the production of GFP increased. Scale bar = 10 *μ*m, (c) dose-dependent response. The cells were exposed to the various concentrations of P-factor and were incubated for 24 hours after the addition of P-factor. Filled circle depicts OSP210-2 (*sxa2*Δ, pAL7-U*mam2*-GFP-LPI) and filled square depicts OSP210-17 (*sxa2*Δ, pAL7-U*sxa2*-GFP-DSPBC4.01). *Y*-axis denotes a percentage of the maximum response. The values were the means of triplicate determinations from a typical experiment.

We explored the time-dependent GFP production of the strains harboring pAL7-U*mam2*-GFP-LPI (OSP210-2) or pAL7-U*sxa2*-GFP-DSPBC4.01 (OSP210-17). The strains were exposed to 1 or 0 *μ*M of P-factor under nitrogen starvation (Figure 2(a)). The strain OSP210-2 exhibited the response with not only the intensity but also the rapidity, which enabled it to discriminate the positive reaction within 3 h after the addition of P-factor. However, the cells had produced measurable GFP protein without the addition of P-factor. On the contrary, pAL7-U*sxa2*-GFP-DSPBC4.01 had caused the cells to produce little GFP protein in the absence of P-factor. The maximum value was relatively high, and the positive signal could be discriminated within 5 h after the addition of P-factor. These results corresponded to the observation with the fluorescent microscope (Figure 2(b)). In both strains, the luminous cells began to appear at the time when the GFP signal was detected by the fluorescent spectrometer. And the elongation of the cell body to form shmoos was also observed, which was the typical response induced by the reception of P-factor [23]. Curiously, the strain OSP210-2 had begun to elongate about two hours earlier than the strain OSP210-17. We also explored the dose-dependent GFP production (Figure 2(c)). The response formed sigmoid curve clearly indicated that the Mam2-P-factor interaction was quantitatively measurable by these strains. Their EC50 values did not show a significant difference between the strains. It was suggested that the efficiency of the subsequent signal transduction pathway had to remain hardly affected by increasing the copy number of plasmids. 

### 3.2. The *cyr1*Δ Strain with Reporter Plasmid

**Figure 3:**
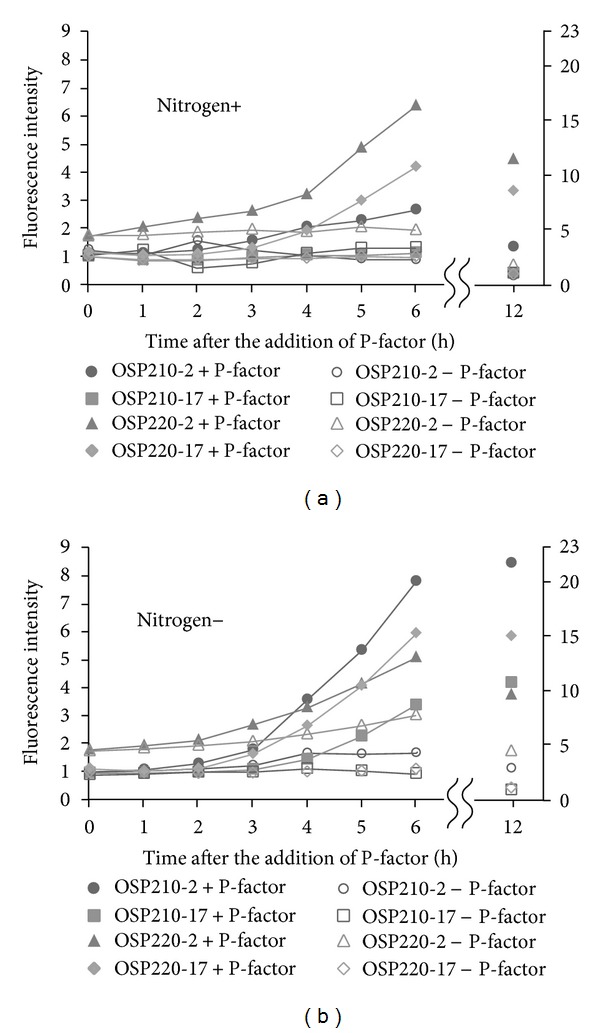
Comparison between *cyr1*Δ and *cyr1*+ strains harboring the reporter plasmid. The cells were exposed to 1 *μ*M of P-factor in the presence of nitrogen (a) and the absence of nitrogen (b) and were taken every hour (0–6 h) or six hours (6–12 h). Filled circle depicts OSP210-2 (*sxa2*Δ, *cyr1*+, pAL7-U*mam2*-GFP-LPI) +P-factor, open circle depicts OSP210-2 −P-factor, filled square depicts OSP210-17 (*sxa2*Δ, *cyr1*+, pAL7-U*sxa2*-GFP-DSPBC4.01) +P-factor, open square depicts OSP210-17 −P-factor, filled triangle depicts OSP220-2 (*sxa2*Δ, *cyr1*Δ, pAL7-U*mam2*-GFP-LPI) +P-factor, open triangle depicts OSP220-2 −P-factor, filled diamond shape depicts OSP220-17 (*sxa2*Δ, *cyr1*Δ, pAL7-U*sxa2*-GFP-DSPBC4.01) +P-factor, and open diamond shape depicts OSP220-17 −P-factor. The values were the means of triplicate determinations from a typical experiment.

The adenylyl cyclase encoded by *cyr1* gene is often deleted from the assay in the mating pathway. The *cyr1*Δ strain exhibits constitutive starvation regardless of the existence of nitrogen and carbon [32]. Being ready to receive the ligands at any time has an advantage in the mating pathway of the assay system, so we examined the effect of *cyr1*-deletion with reporter plasmids. The cells were exposed to 1 or 0 *μ*M of P-factor under the presence or absence of nitrogen, and the time-dependent responses were measured (Figure 3). Under the presence of nitrogen, the *cyr1*Δ strain harboring pAL7-U*mam2*-GFP-LPI (OSP220-2) responded most intensely at 12 h. Compared to the strain without P-factor, the difference in GFP production began to appear at 3 h and was completely distinguishable at 4 h and beyond. However, these strains had already exhibited high background at 0 h. On the other hand, the *cyr1*Δ strain harboring pAL7-U*sxa2*-GFP-DSPBC4.01 (OSP220-17) became distinguishable at 4 h. In addition, the strain without P-factor did not produce GFP protein at all. Unsurprisingly, the *cyr1*+ strain harboring pAL7-U*sxa2*-GFP-DSPBC4.01 (OSP210-17) with or without P-factor did not show the response in the presence of nitrogen. Interestingly, the *cyr1*+ strain harboring pAL7-U*mam2*-GFP-LPI (OSP210-2) could respond to P-factor by 12 h having passed even under the presence of nitrogen. Under the absence of nitrogen, the *cyr1*Δ strain OSP220-2 was with high background and began to produce GFP at about 3 h as is the case in the presence of nitrogen. However, this strain did not raise the response as we had expected. The *cyr1*Δ strain OSP220-17 became distinguishable about one hour earlier than *cyr1*+ strain OSP210-17, and the intensity of the response was also increased. The *cyr1*-deletion was quite effective in the strain OSP220-17 regardless of the existence of the nitrogen.

### 3.3. The Ectopic *mam2* Expression in the Strain Harboring Reporter Plasmid

**Figure 4:**
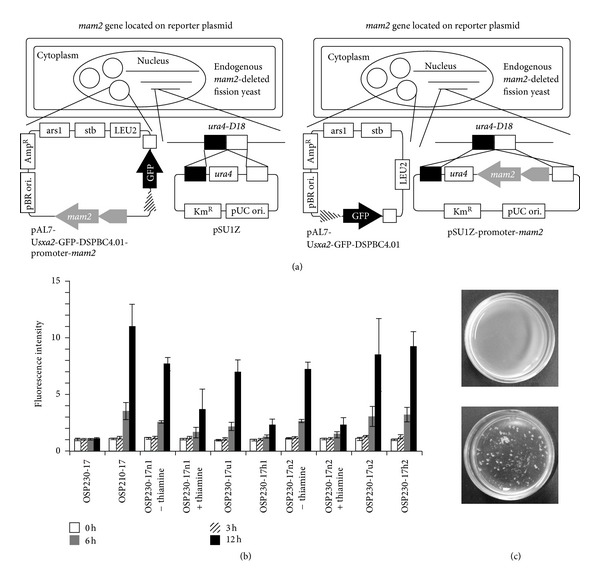
Ectopic *mam2* expression in *mam2*Δ strain. (a) The schematic strategy of ectopic *mam2* expression in endogenous *mam2*Δ strain. Left, *mam2* gene (gray arrow) was driven on reporter plasmid. To complement *ura4* gene, empty pSU1Z vector was introduced into *ura4* locus on chromosome. Right, *mam2* gene on pSU1Z vector was introduced into the *ura4* locus. The gray pentagon depicted the promoter, which was *nmt1* promoter, *urg1* promoter or hCMV promoter. The two plasmids (reporter and receptor) were transformed at the same time. (b) The *mam2* gene was expressed under the control of *nmt1* promoter, *urg1* promoter or hCMV promoter on chromosome or the reporter plasmid. OSP210-17 was used as a positive control. OSP230-17 was used as a negative control and did not express *mam2* gene under the control of any promoter. The cells were exposed to 1 *μ*M of P-factor and were incubated for 0 h (open box), 3 h (shaded box), 6 h (gray box), or 12 h (filled box) after the addition of P-factor. The strains including the *nmt1* promoter were assayed with 0 (on) or 15 (off) *μ*M of thiamine. The *urg1* promoter was constitutively activated under the nitrogen starvation. The values were the means of triplicate determinations from a typical experiment. The error bars represent ± standard error. (c) Upper, the preculture medium of the strain OSP230-17 h2 expressing *mam2* gene under the control of chromosomal hCMV promoter with pAL7-U*sxa2*-GFP-DSPBC4.01. Lower, the preculture medium of the strain OSP230-2 h2 expressing *mam2* gene under the control of chromosomal hCMV promoter with pAL7-U*mam2*-GFP-LPI.

As a model for heterologous GPCR assay with the reporter plasmid, the *mam2* gene was ectopically expressed under the control of other promoters in the endogenous *mam2*Δ strain, OSP230 (*h*−, *leu1-32*, *ura4-D18*, *sxa2*Δ, *mam2*Δ). Three promoters were used to express the *mam2* gene; the first is no message in thiamine 1 (*nmt1*) promoter, which is the most common inducible promoter and exhibits the strongest expression in the fission yeast [33, 34]; the second is uracil regulatable 1 (*urg1*) promoter, which is an inducible promoter and expressed moderately. While *urg1* promoter is repressed by the removal of uracil from the medium, it was maximally induced by either the addition of the uracil or the removal of the nitrogen [28]; the third is human cytomegalovirus (hCMV) promoter which is the strong promoter activated constitutively [35]. The *mam2* gene was driven on either of the chromosomal integration vectors for fission yeast, pSU1Z vector (or its derivatives) or the reporter plasmid (Figure 4(a)). The transformation to introduce the receptor and the reporter was performed at the same time. When the parental strain OSP230 was co-transformed by 150 ng of the reporter plasmid and 150 ng of the linearized integration vector, 37 (SD ± 14) colonies appeared. Exposed to 1 *μ*M of P-factor, the response could be adequately detected in 5 *mam2* expression patterns out of 6, which fell slightly below that in the native expression pattern (Figure 4(b)). Perhaps too much accumulation of Mam2 protein might hinder the GFP production in the strain OSP230-17h1 whose *mam2* was constitutively expressed under the control of hCMV promoter on the high copy plasmid. On the other hand, the GFP production was not inhibited by the constitutive chromosomal expression whose *mam2* expression was smaller than episomal expression. The induced *nmt1* promoter shows little difference between the episomal and chromosomal expression. Under this experimental condition in which the cells were pre-incubated for 20 h, the *mam2* gene was strongly expressed by induced *nmt1* promoter for 2–5 h after the intracellular thiamine was completely carried out of the cell body. Regulating the pre-incubation time might improve the response. In spite of the addition of thiamine, the *nmt1* promoter was not completely repressed. This result coincided with the previous data [28]. The *urg1* promoter did not show a significant difference between the episomal and chromosomal expression. In both strains, it was very easy to operate *urg1* promoter since that was automatically repressed and induced under this experimental condition. Although we followed the same procedure with pAL7-U*mam2*-GFP-LPI as we had with pAL7-U*sxa2*-GFP-DSPBC4.01, the assay had gone wrong. The reason why we could not assay was that the cells formed aggregates that did not respond to P-factor at all during the preculture (Figure 4(c)).

## 4. Discussion

### 4.1. New Reporter System

The reporter system constructed in this study was considerably tractable. These reporter plasmids were easily transformed with high efficiency even at the same time as the chromosomal integration vector was introduced. In addition, the plasmid is so flexible that it is easy to reconstruct; for example, the selection marker could be changed depending on the genotype of laboratory stock strains. Moreover, as enough GFP for detection was produced in this assay system, the reporter gene did not have to be an intense one such as lacZ or luciferase, which required the substrate degradation at the detection for sensitive readout. As a reporter region of the plasmid, we concluded that a combination of *sxa2* upstream region and the SPBC4.01 downstream region was the most suitable due to the best SNR. The cells responding to P-factor began to appear at 5 h and became obviously luminous at 6 h, while few luminous cells appeared for 24 h in the absence of P-factor. The combination of *mam2* upstream region and LPI terminator responded most rapidly and intensely of all, and began to respond at 3 h and became absolutely distinguishable at 4 h. However, we were not able to recommend using the reporter region for two reasons: firstly because of the excessively high background (many luminous cells appeared within 8 h and SNR was not so good compared to others), and secondly because nonspecific aggregation was readily formed.

### 4.2. The Combination of the Reporter Plasmid and *cyr1*-Deletion

In the combination of pAL7-U*sxa2*-GFP-DSPBC4.01, the *cyr1*-deletion had both positive and negative aspects. The positive aspects were that the assay medium was not limited and that the cells responded a few hours earlier. The negative aspects were that the *cyr1*Δ strains became more difficult to deal with; for example, it took a longer time to divide (maximum doubling time of OSP210-17 →OSP220-17: 3.20 h → 4.76 h at 30°C in EMM) and the transformation efficiency became significantly lower (about one hundredth). In addition to those demerits, what was worse was what occurred in the *cyr1*Δ strain harboring pAL7-U*mam2*-GFP-LPI. Under nitrogen starvation, the *cyr1*Δ strain responded less intensely than the *cyr1*+ strain. One explanation could be that there was a difference in the optimal pre-incubation time to maximally respond to P-factor between the strain OSP220-2 and other strains. It was implied that in order to retain pAL7-U*mam2*-GFP-LPI, the intracellular condition had to be slightly affected (described below). 

### 4.3. Ectopic *mam2* Expression

As a model for the heterologous GPCRs, we expressed *mam2* gene under the control of three kinds of promoters in the *mam2*Δ strain. It was fundamentally convenient for the associated experiments to express the receptor gene on the reporter plasmid, although the huge receptor expression could cause problems in the response. The expression by the *nmt1* and *urg1* promoter on the high copy plasmid had little impact on the reception of P-factor, but the reduction of response was seen in the strain whose *mam2* gene was constitutively expressed by hCMV promoter on the high copy plasmid. It was suggested that too much Mam2 production presumably caused the unfolded protein response, which had a harmful effect on the cell [36]. Thus, it would be indispensable to repress the heterologous GPCR expression during mitosis. In fact, *nmt1* promoter could reproduce the response well in spite of demonstrating stronger expression than hCMV promoter. When the assay with *urg1* promoter was performed, the operations were very easy because the cells were automatically repressed and induced in this system. However, note that the induction of *urg1* promoter can depend on the intracellular condition [28], so the *cyr1*Δ strain might be unable to repress the expression of *urg1* promoter as well as the *cyr1*+ strain.

### 4.4. Intracellular Effect of pAL7-U*mam2*-GFP-LPI

The Cyr1 protein is required through the nutrient pathway to perceive not nitrogen but rather a carbon resource [37]. In fact, the *cyr1*-deletion makes the cell unable to perceive the carbon resource. However, carbon starvation does not result in the autophagy for meiosis, which is induced by nitrogen starvation [38]. The reason why the *cyr1*-deletion (carbon starvation mimic) affects the mating pathway is presumably because it facilitates expression of the *ste11* gene which commonly plays a key role in several intracellular signal transduction pathways including not only mating pathway but also nutrition signaling and stress signaling pathways [30, 37]. In this study, there was some circumstantial evidence suggesting that such the *ste11*-induction was seen in the *cyr1*+ strain harboring pAL7-U*mam2*-GFP-LPI (OSP210-2). The strain OSP210-2 could respond to P-factor even under the existence of nitrogen (Figure 3). Furthermore, the morphological change made progress about two hours earlier than that of the strain OSP210-17 (Figure 2(b)). The promoter and/or activating regions must be related to the GFP production, but the regions are unlikely to have an effect on the morphological change. It was suggested that DNA and/or RNA derived from pAL7-U*mam2*-GFP-LPI made *ste11* gene more likely to be induced as is often the case with stress response and nutrient starvation.

## Figures and Tables

**Table 1:** Fission yeast strains used in this study.

Strain	Genotype
OSP210	*h*−, *leu1-32*, *ura4-D18*, *sxa2*Δ
OSP210-0	*h*−, *leu1-32*, *ura4-D18*, *sxa2*Δ, pSU1Z, pAL7
OSP210-1	*h*−, *leu1-32*, *ura4-D18*, *sxa2*Δ, pSU1Z, pAL7-U*dhc1*-GFP-LPI
OSP210-2	*h*−, *leu1-32*, *ura4-D18*, *sxa2*Δ, pSU1Z, pAL7-U*mam2*-GFP-LPI
OSP210-3	*h*−, *leu1-32*, *ura4-D18*, *sxa2*Δ, pSU1Z, pAL7-U*mam3*-GFP-LPI
OSP210-4	*h*−, *leu1-32*, *ura4-D18*, *sxa2*Δ, pSU1Z, pAL7-U*rgs1*-GFP-LPI
OSP210-5	*h*−, *leu1-32*, *ura4-D18*, *sxa2*Δ, pSU1Z, pAL7-USPBC4.01-GFP-LPI
OSP210-6	*h*−, *leu1-32*, *ura4-D18*, *sxa2*Δ, pSU1Z, pAL7-U*spk1*-GFP-LPI
OSP210-7	*h*−, *leu1-32*, *ura4-D18*, *sxa2*Δ, pSU1Z, pAL7-U*sxa2*-GFP-LPI
OSP210-8	*h*−, *leu1-32*, *ura4-D18*, *sxa2*Δ, pSU1Z, pAL7-U*dhc1*-GFP-D*dhc1 *
OSP210-9	*h*−, *leu1-32*, *ura4-D18*, *sxa2*Δ, pSU1Z, pAL7-U*mam2*-GFP-D*mam2 *
OSP210-10	*h*−, *leu1-32*, *ura4-D18*, *sxa2*Δ, pSU1Z, pAL7-U*mam3*-GFP-D*mam3 *
OSP210-11	*h*−, *leu1-32*, *ura4-D18*, *sxa2*Δ, pSU1Z, pAL7-U*rgs1*-GFP-D*rgs1 *
OSP210-12	*h*−, *leu1-32*, *ura4-D18*, *sxa2*Δ, pSU1Z, pAL7-USPBC4.01-GFP-DSPBC4.01
OSP210-13	*h*−, *leu1-32*, *ura4-D18*, *sxa2*Δ, pSU1Z, pAL7-U*spk1*-GFP-D*spk1 *
OSP210-14	*h*−, *leu1-32*, *ura4-D18*, *sxa2*Δ, pSU1Z, pAL7-U*sxa2*-GFP-D*sxa2 *
OSP210-15	*h*−, *leu1-32*, *ura4-D18*, *sxa2*Δ, pSU1Z, pAL7-U*mam2*-GFP-DSPBC4.01
OSP210-16	*h*−, *leu1-32*, *ura4-D18*, *sxa2*Δ, pSU1Z, pAL7-U*mam3*-GFP-DSPBC4.01
OSP210-17	*h*−, *leu1-32*, *ura4-D18*, *sxa2*Δ, pSU1Z, pAL7-U*sxa2*-GFP-DSPBC4.01
OSP220	*h*−, *leu1-32*, *ura4-D18*, *sxa2*Δ, *cyr1*Δ
OSP220-0	*h*−, *leu1-32*, *ura4-D18*, *sxa2*Δ, *cyr1*Δ, pSU1Z, pAL7
OSP220-2	*h*−, *leu1-32*, *ura4-D18*, *sxa2*Δ, *cyr1*Δ, pSU1Z, pAL7-U*sxa2*-GFP-DSPBC4.01
OSP220-17	*h*−, *leu1-32*, *ura4-D18*, *sxa2*Δ, *cyr1*Δ, pSU1Z, pAL7-U*mam2*-GFP-LPI
OSP230	*h*−, *leu1-32*, *ura4-D18*, *sxa2*Δ, *mam2*Δ,
OSP230-17	*h*−, *leu1-32*, *ura4-D18*, *sxa2*Δ, *mam2*Δ, pSU1Z, pAL7-U*sxa2*-GFP-DSPBC4.01
OSP230-17n1	*h*−, *leu1-32*, *ura4-D18*, *sxa2*Δ, *mam2*Δ, pSU1Z, pAL7-U*sxa2*-GFP-DSPBC4.01-P*nmt1*-*mam2 *
OSP230-17u1	*h*−, *leu1-32*, *ura4-D18*, *sxa2*Δ, *mam2*Δ, pSU1Z, pAL7-U*sxa2*-GFP-DSPBC4.01-P*urg1*-*mam2 *
OSP230-17h1	*h*−, *leu1-32*, *ura4-D18*, *sxa2*Δ, *mam2*Δ, pSU1Z, pAL7-U*sxa2*-GFP-DSPBC4.01-PhCMV-*mam2 *
OSP230-17n2	*h*−, *leu1-32*, *ura4-D18*, *sxa2*Δ, *mam2*Δ, pSU1Z-Pnmt1-*mam2*, pAL7-U*sxa2*-GFP-DSPBC4.01
OSP230-17u2	*h*−, *leu1-32*, *ura4-D18*, *sxa2*Δ, *mam2*Δ, pSU1Z-Purg1-*mam2*, pAL7-U*sxa2*-GFP-DSPBC4.01
OSP230-17h2	*h*−, *leu1-32*, *ura4-D18*, *sxa2*Δ, *mam2*Δ, pSU1Z-PhCMV-*mam2*, pAL7-U*sxa2*-GFP-DSPBC4.01
OSP230-2h2	*h*−, *leu1-32*, *ura4-D18*, *sxa2*Δ, *mam2*Δ, pSU1Z-PhCMV-*mam2*, pAL7-U*mam2*-GFP-LPI

**Table 2:** The reporter plasmids and the GFP production of those transformants.

Plasmid name	Reporter region of reporter plasmid			Resultant strain	Fluorescence intensity at			SNR^c^
	upstream	reporter	downstream		0 h	24 h (−)^a^	24 h (+)^b^	
pAL7-U*dhc1*-GFP-LPI	*dhc1 *	GFP	LPI	OSP210-1	1.07 (±0.12)	1.02 (±0.03)	1.05 (±0.04)	1.03
pAL7-U*mam2*-GFP-LPI	*mam2 *	GFP	LPI	OSP210-2	1.14 (±0.10)	4.05 (±0.43)	28.62 (±5.74)	7.07
pAL7-U*mam3*-GFP-LPI	*mam3 *	GFP	LPI	OSP210-3	1.05 (±0.10)	1.02 (±0.10)	7.66 (±2.21)	7.51
pAL7-U*rgs1*-GFP-LPI	*rgs1 *	GFP	LPI	OSP210-4	1.52 (±0.14)	1.88 (±0.23)	4.12 (±0.98)	2.19
pAL7-USPBC4.01-GFP-LPI	SPBC4.01	GFP	LPI	OSP210-5	1.06 (±0.13)	1.09 (±0.06)	3.75 (±2.64)	3.44
pAL7-U*spk1*-GFP-LPI	*spk1 *	GFP	LPI	OSP210-6	1.15 (±0.08)	1.58 (±0.18)	3.71 (±0.74)	2.35
pAL7-U*sxa2*-GFP-LPI	*sxa2 *	GFP	LPI	OSP210-7	1.05 (±0.09)	1.05 (±0.09)	9.82 (±2.09)	9.35
pAL7-U*dhc1*-GFP-D*dhc1 *	*dhc1 *	GFP	*dhc1 *	OSP210-8	1.07 (±0.12)	1.02 (±0.03)	1.06 (±0.05)	1.04
pAL7-U*mam2*-GFP-D*mam2 *	*mam2 *	GFP	*mam2 *	OSP210-9	1.04 (±0.13)	1.15 (±0.20)	6.46 (±1.06)	5.62
pAL7-U*mam3*-GFP-D*mam3 *	*mam3 *	GFP	*mam3 *	OSP210-10	1.05 (±0.13)	0.99 (±0.11)	4.02 (±1.68)	4.06
pAL7-U*rgs1*-GFP-D*rgs1 *	*rgs1 *	GFP	*rgs1 *	OSP210-11	1.44 (±0.09)	2.36 (±0.90)	7.96 (±3.27)	3.37
pAL7-USPBC4.01-GFP-DSPBC4.01	SPBC4.01	GFP	SPBC4.01	OSP210-12	1.05 (±0.08)	1.03 (±0.31)	7.84 (±1.85)	7.61
pAL7-U*spk1*-GFP-D*spk1 *	*spk1 *	GFP	*spk1 *	OSP210-13	1.17 (±0.15)	1.25 (±0.03)	4.64 (±1.31)	3.71
pAL7-U*sxa2*-GFP-D*sxa2 *	*sxa2 *	GFP	*sxa2 *	OSP210-14	1.12 (±0.17)	1.03 (±0.06)	8.43 (±2.91)	8.18
pAL7-U*mam2*-GFP-DSPBC4.01	*mam2 *	GFP	SPBC4.01	OSP210-15	0.99 (±0.02)	1.21 (±0.15)	6.99 (±3.19)	5.78
pAL7-U*mam3*-GFP-DSPBC4.01	*mam3 *	GFP	SPBC4.01	OSP210-16	1.03 (±0.03)	0.99 (±0.08)	6.40 (±2.96)	6.46
pAL7-U*sxa2*-GFP-DSPBC4.01	*sxa2 *	GFP	SPBC4.01	OSP210-17	0.99 (±0.04)	1.06 (±0.05)	15.54 (±3.09)	14.66

Each value of fluorescence intensity was mean (±SD) from more than three independent experiments. ^a^(−): incubation without P-factor. ^b^(+): incubation with P-factor. ^c^SNR: signal to noise ratio, SNR was obtained by dividing the fluorescence intensity at 24 h (+) by that at 24 h (−).

**Table 3:** Primer information.

Primer name	Target gene	Region^a^	Forward (5′→3′)	Reverse (5′→3′)
pAL7inv			GAGCAAAAGGCCAGCAAAAG	AACCGTATTACCGCCTTTGA
GFPORF			ATGGGCGTGATCAAGCCCG	TTAGCCGGCCTGGCGGGGT
*dhc1*up	SPAC1093.06c/*dhc1 *	−1044	AAGCACGCGCTCTAATTCAT	GGTGTCAAGAAAACTTGACCG
*dhc1*dw		+958	AACTTGAAACTATTTGTTGTTTACTA	GAATCTGAGGTTGATGTTGAA
*mam2*up	SPAC11H11.04/*mam2 *	−1068	CATCGGGATTGCATTGAGAGT	AATGTCAGAGGGAGCAAGAACA
*mam2*dw		+1010	CTTACGCCTGAATGTATCTTT	ACTCAAAGCCATAACTGTGC
*mam3*up	SPAP11E10.02c/*mam3 *	−1041	TTTTAGAAAGTGTCTATTGTACC	GACGAATTATGGGAAGATCAAG
*mam3*dw		+997	ATAAAGTTAATGTTTTATATTTATTTTACA	ACTGAGAATGTCGTCTGTCC
*rgs1*up	SPAC22F3.12c/*rgs1 *	−1118	GGCAGGTGTAAGAAGCGTTG	CCAAAGCTGATTCTTACTTTTACGA
*rgs1*dw		+944	TGCATAGAAAACAATCGTGT	CGAAAGAATCCTGCTGTTAC
SPBC4.01up	SPBC4.01	−985	CCCATCTGGGTGAAAGAGTG	CCATTCTTAAACCGTAATTTTAAATTG
SPBC4.01dw		+992	ACAAACATAAATAAGATTTTGTAAAC	AATTATTGCTGTCGCCGAAC
*spk1*up	SPAC31G5.09c/*spk1 *	−1040	GGACGCCAAGGGAAATTTAT	TAGACTACAAATTGAAAAACTTGAAAG
*spk1*dw		+958	AAAGCTTCAACTAGAATTCTCCT	CAACCGATGACGGTATTTAT
*sxa2*up	SPAC1296.03c/*sxa2 *	−1363	AGATTATGGGGTAGTGGGTTC	GCATTGAAAAGAGAGACAATGA
*sxa2*dw		+945	AAGTTTAATATCGGAAAATTTAA	CGGAAGTTAGGCTTGTGTGC

^a^Region represents how distant from ATG or stop codon of its target gene is ORF.
